# Automated multi-dose dispensing in persons with and without Alzheimer’s disease—impacts on pharmacotherapy

**DOI:** 10.1007/s00228-021-03258-y

**Published:** 2021-11-27

**Authors:** Sanna Vallius, Heidi Taipale, Marjaana Koponen, Anna-Maija Tolppanen, Antti Tanskanen, Sirpa Hartikainen, Miia Tiihonen

**Affiliations:** 1 School of Pharmacy, University of Eastern Finland, P.O. Box 1627, 70211 Kuopio, Finland; 2 Kuopio Research Centre of Geriatric Care, University of Eastern Finland, Kuopio, Finland; 3 Department of Forensic Psychiatry, University of Eastern Finland, Niuvanniemi Hospital, Kuopio, Finland; 4 Department of Clinical Neuroscience, Karolinska Institutet, Stockholm, Sweden; 5 Center for Psychiatry Research, Stockholm City Council, Stockholm, Sweden; 6 Centre for Medicine Use and Safety, Faculty of Pharmacy and Pharmaceutical Sciences, Monash University, Melbourne, VIC Australia

**Keywords:** Multi-dose dispensing, Alzheimer’s disease, Dementia, Psychotropic drugs, Potentially inappropriate medications, Polypharmacy

## Abstract

**Purpose:**

We investigated the drug use before and after transition to automated multi-dose dispensing (MDD) service among persons with Alzheimer’s disease (AD) and compared whether the changes were similar in persons without AD.

**Methods:**

The register-based Finnish nationwide MEDALZ cohort includes 70,718 community-dwelling persons diagnosed with AD during 2005–2011. Each person who initiated MDD was matched in both groups with a comparison person without MDD by age, gender and for persons with AD, also time since AD diagnosis at the start of MDD. The study cohort included 15,604 persons with AD in MDD and 15,604 no-MDD, and 5224 persons without AD in MDD and 5224 no-MDD. Point prevalence of drug use was assessed every 3 months, from 1 year before to 2 years after the start of MDD and compared between persons in MDD to those who did not have MDD.

**Results:**

MDD was started on average 2.9 (SD 2.1) years after AD diagnosis. At the start of MDD, the prevalence of drug use increased especially for antipsychotics, antidepressants, opioids, paracetamol and use of ≥ 10 drugs among persons with and without AD. Prevalence of benzodiazepine use (from 12% 12 months before to 17% at start of MDD), memantine (from 29 to 46%) and ≥ 3 psychotropics (from 3.2 to 6.0%) increased among persons with AD. Decreasing trend was observed for benzodiazepine-related drugs, urinary antispasmodics and non-steroidal anti-inflammatory drugs.

**Conclusion:**

MDD seems to be initiated when use of psychotropics is initiated and the number of drugs increases.

**Supplementary information:**

The online version contains supplementary material available at 10.1007/s00228-021-03258-y.

## Introduction

The automated multi-dose dispensing (MDD) is a health technology aimed at helping with daily self-administration of drugs. The service was launched in Finland in 2002 and is widely used in Nordic countries and the Netherlands [1–3]. In MDD service, patient’s regularly used drugs are machine-packed in plastic unit-dose pouches according to time of administration [4, 5]. The unit-dose pouches are dispended to patient from the pharmacy every fortnight. Ministry of Social Affairs (2016) states that MDD aims to promote drug safety, adherence and to decrease medication costs [5]. It is commonly used in home care [6] and residential care to reduce nurses’ working time on drug administration [5].

In previous studies, MDD has been associated with polypharmacy, potentially inappropriate drug (PIM) use and unnecessary drug use [7–10]. However, MDD is important for older persons with inabilities to manage their medication. According to the Finnish guideline, the medication regimen should be reviewed by both physician and pharmacist at the start of MDD and regularly during its use [5]. In the interprofessional medication assessment, pharmacists review the doses, drug interactions and PIMs, while physicians have the professional responsibility to make decisions on the drug treatment and prescribe drugs which are to be administered in MDD service and monitor the impact of the drug treatment, usually in co-operation with a nurse or caretaker. This interprofessional collaboration should solve drug-related problems and implement medication changes [5, 11]. According to previous studies, a significant proportion (24–33%) of MDD service users have Alzheimer’s disease (AD) in Finland [10, 12]. Persons with AD experience cognitive and neuropsychiatric symptoms (NPS) with the disease often leading to deficits in instrumental activities of daily living, including the ability to manage drug treatment [13, 14]. However, no previous study has addressed MDD among persons with AD and its impact on prevalence of specific medication use.

The aim of this study among persons with AD was to investigate drug use before and after MDD and in comparison to matched persons not using MDD. The same analyses were also conducted among persons without AD.

## Methods

### Study population

This study used data from the MEDALZ study which includes 70,718 community-dwelling persons who were diagnosed with AD in Finland during 2005–2011 [15]. Persons with AD were identified from the Special Reimbursement Register maintained by the Social Insurance Institution (SII). The register includes persons entitled for special reimbursement of drugs due to certain chronic diseases. The Finnish Current Care Guideline on cognitive disorders recommends that all persons with AD having no contraindications should be treated with antidementia drugs [16]. In order to get special reimbursement for AD drugs, persons had to meet clinical diagnosis criteria based on the NINCDS-ADRDA and DSM-IV criteria [15, 17, 18]. The diagnostic process also included computed tomography or magnetic resonance imaging, exclusion of alternative diagnosis and a confirmation of diagnosis of AD by a neurologist or geriatrician.

A matched comparison cohort was formed of persons without AD, according to age, gender and region of hospital district. Data for persons with and without AD were extracted from the Finnish nationwide healthcare registers, including the Prescription Register (1995–2015), the Special Reimbursement Register (1972–2015), Causes of death register from Statistics Finland (2005–2015) and the Care Register for Health Care.

### Multi-dose dispending service and matching

**Fig. 1:**
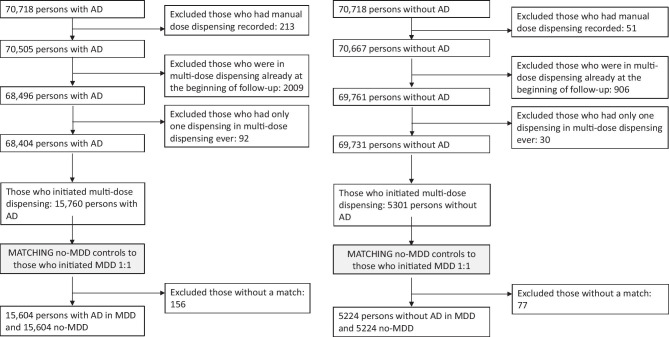
Flowchart of study selection. AD, Alzheimer’s disease; MDD, multi-dose dispensing; no-MDD, persons without multi-dose dispensing

Patients who initiated MDD service use were identified from the Prescription Register based on a specific code indicating that drug has been dispensed by MDD service. The first appearance of this was defined as initiation of MDD. Exclusion criteria for this study were as follows: indication of manual dose dispensing in drug purchases, having MDD already at the date of cohort entry (date of AD diagnoses or corresponding date for persons without AD) and having only one dispensing in multi-dose dispensing ever (which may have been a mistake made by the pharmacy). MDD initiations were selected between AD diagnoses/corresponding date for non-AD persons and death/end of data linkage (31 December 2015). Exclusions are described in more detail in Fig. 1. Each person for whom MDD was initiated was matched with a comparison person without MDD by age (± 3 years), gender, AD, region of residence (hospital district) and time since AD diagnosis (± 0.5 years) at the start of MDD. The comparison person was required to have recorded drug dispensing during 6 months before the matching date to ensure that they were community-dwelling and not institutionalized (to an institution providing drugs and not recorded in the Prescription Register). Persons without a match *n* = 156 with AD and *n* = 77 without AD were excluded.

### Drug exposure

The drugs were identified from the Prescription Register according to the WHO Anatomical Therapeutic Chemical (ATC) classification system [19]. Over-the-counter drugs were not included into the study. The number of drugs used was calculated as the sum of all reimbursed drugs used using 5th ATC code level. The following drugs/drug classes were analysed according to MDD (see Online resource Table 1): antidementia drugs, benzodiazepines, benzodiazepine-related drugs, antipsychotics, antidepressants, opioids, paracetamol, non-steroidal anti-inflammatory drugs, oral antidiabetics, loop diuretics, other diuretics, beta blockers, calcium channel blockers, renin-angiotensin group, statins, antiepileptics, proton pump inhibitors (PPIs) and urinary antispasmodics excluding mirabegron.

**Table 1:** Characteristics of the study cohort according to Alzheimer’s disease (AD) and multi-dose dispensing (MDD) status

	**Persons with AD**			**Persons without AD**		
	**MDD** ***N* = 15,604** ***N* (%)**	**no-MDD** ***N* = 15,604** ***N* (%)**	***p* value**	**MDD** ***N* = 5224** ***N* (%)**	**no-MDD** ***N* = 5224** ***N* (%)**	***p* value**
**Cohort characteristics**						
Female sex	11487 (73.6)	11487 (73.6)	1.00	3912 (74.9)	3912 (74.9)	1.00
Age, years			0.030			0.003
≤ 69	476 (3.1)	466 (3.0)		30 (0.6)	30 (0.6)	
70–79	3709 (23.8)	3692 (23.7)		466 (8.9)	477 (9.1)	
80–89	9469 (49.5)	9657 (50.5)		3215 (64.6)	3375 (61.5)	
≥ 90	1950 (12.5)	1789 (11.5)		1513 (29.0)	1342(25.7)	
Mean time since AD diagnosis, years	2.9 (± 2.1)	2.9 (± 2.1)	0.974	4.2 (± 2.4)	4.2 (± 2.4)	0.957
**Comorbidities**						
Any cardiovascular disease	9620 (61.7)	9061 (58.1)	< 0.001	3686 (70.6)	3168 (60.6)	< 0.001
Hypertension	6829 (43.8)	6345 (40.7)	< 0.001	2645 (50.6)	2293 (43.9)	< 0.001
Coronary disease	4444 (28.5)	4113 (26.4)	< 0.001	1734 (33.2)	1382 (26.5)	< 0.001
Chronic heart failure	1919 (12.3)	1760 (11.3)	0.005	989 (18.9)	558 (10.7)	< 0.001
Atrial fibrillation	2130 (13.7)	2016 (12.9)	0.057	868 (16.6)	574 (11.0)	< 0.001
Diabetes	3381 (21.7)	2991 (19.2)	< 0.001	1384 (26.5)	959 (18.4)	< 0.001
Osteoporosis	3198 (20.5)	2952 (18.9)	< 0.001	1308 (25.0)	965 (18.5)	< 0.001
Hypothyreosis	2503 (16.0)	2327 (14.9)	0.006	952 (18.2)	827 (15.8)	0.001
Glaucoma	1950 (12.5)	1876 (12.0)	0.202	859 (16.4)	837 (16.0)	0.559
Any cancer	1818 (11.7)	1732 (11.1)	0.125	648 (12.4)	604 (11.6)	0.185
Asthma/COPD	1599 (10.2)	1570 (10.1)	0.587	682 (13.1)	460 (8.8)	< 0.001
Stroke	1581 (10.1)	1346 (8.6)	< 0.001	681 (13.0)	365 (7.0)	< 0.001
Any psychiatric disorder	824 (5.3)	651 (4.2)	< 0.001	361 (6.9)	187 (3.6)	< 0.001
Rheumatoid arthritis	746 (4.8)	755 (4.8)	0.812	307 (5.9)	224 (4.3)	< 0.001
Substance abuse	521 (3.3)	338 (2.2)	< 0.001	161 (3.1)	62 (1.2)	< 0.001
Epilepsy	312 (2.0)	251(1.6)	0.009	114 (2.2)	67 (1.3)	< 0.00
**Any hospital care during previous 1 month (%)**	15.6	11.5		18.87	7.4	
**Socioeconomic positions**			< 0.001			< 0.001
High	4850 (31.1)	5358 (34.3)		1608 (30.8)	1896 (36.3)	
Medium	9475 (60.7)	9146 (58.6)		2875 (55.0)	2877 (55.1)	
Low	1110 (7.1)	949 (6.1)		462 (8.8)	244 (4.7)	
Unknown	169 (1.1)	156 (1.0)		279 (5.3)	207 (4.0)	
**Mean total number of drugs (SD)**						
One month before MDD	6.8 (± 3.2)	6.1 (± 3.2)	0.546	7.5 (± 3.3)	5.4 (± 3.2)	0.050
At start of MDD	7.4 (± 3.1)	6.2 (± 3.2)	< 0.001	8.2 (± 3.1)	5.5 (± 3.2)	0.094
One year after start of MDD	7.2 (± 3.0)	6.1 (± 3.3)	< 0.001	7.8 (± 3.2)	5.5 (± 3.2)	0.307
Two years after start of MDD	7.0 (± 3.2)	6.1 (± 3.4)	< 0.001	7.7 (± 3.3)	5.4 (± 3.2)	0.204

*COPD* chronic obstructive pulmonary disease; *SD* standard deviation

In addition, we assessed concomitant use of three or more psychotropics and use of ten or more drugs. Psychotropic drugs included antipsychotics, benzodiazepines, benzodiazepine-related drugs and antidepressants.

The drug use was derived from single and combination products which contain two or three active ingredients. Drug use was modelled using mathematical modelling method PRE2DUP [20]. This method estimates duration of drug use (e.g. when drug use started and ended) and dose by considering the purchased amount in defined daily dose (DDD). The method takes into account stockpiling of drugs, personal purchasing patterns and periods of hospital/institutional care when drugs are provided by the caring unit and not recorded in the Prescription Register. For each individual, the start of the MDD was defined as the index date (the corresponding matching date for no-MDD persons). Drug use was evaluated as 2-week point prevalence of use every 3 months, starting from 12 months before and until 24 months after the index date. Two-week period was chosen because drugs in MDD service are typically dispensed for 2 weeks of treatment.

### Other characteristics

Data on comorbidities were identified from the Special Reimbursement Register, Prescription Register and from Care Register for Health Care in terms of cardiovascular disease (hypertension, coronary artery disease, chronic heart failure, atrial fibrillation), diabetes, osteoporosis, hypothyreosis, glaucoma, cancer, asthma/COPD, stroke, psychiatric disorders, rheumatoid arthritis and other connective tissue diseases, substance abuse and epilepsy (see detailed definitions in Online resource Table 2). Socioeconomic position was obtained from Statistics Finland, defined as the highest position recorded in the middle age (45–55 years) and was classified into four categories as high, medium, low and unknown.

### Statistical analyses

The data was analysed comparing prevalences among persons who started MDD to persons who did not have MDD. The prevalences are reported proportions of users from persons who were alive and in outpatient care at each 2-week observation period (e.g. persons were excluded from a specific 2-week time window if the person was hospitalized/institutionalized for more than 5 days of the period or survived < 8 days of the period) and the results were presented with 95% confidence intervals. Comparison persons were censored if they initiated MDD and persons without AD were censored if they were diagnosed with AD. A Pearson’s chi-squared test was used to compare the prevalences of drug use and background variables between persons with MDD to matched no-MDD persons separately for persons with and without AD. The *t*-test was used for continuous variables (time since diagnosis and the number of drugs). The statistical analyses were performed using SPSS 25.0.

According to Finnish legislation, no ethics committee approval was needed for this study because only de-identified data was utilized and there was no need for contact with the cohort members and the permission for the data use was received from the register maintainers.

## Results

### Study participants

Characteristics of persons with and without AD according to MDD status at the index date are presented in Table 1. The study cohort included 15,604 persons with AD in MDD and 15,604 no-MDD, and 5224 persons without AD in MDD and 5224 no-MDD. The majority of persons in MDD were women in both cohorts and the mean age at the start of MDD was 82.7 years among persons with AD and 86.4 years among persons without AD. The mean time since AD diagnosis was 2.9 (SD 2.1) years. Persons with MDD had more comorbid conditions than persons without MDD and also more inpatient hospital days within the first month before the start of MDD. Cardiovascular diseases, diabetes and osteoporosis were the most common comorbidities among persons with AD and without AD.

### Prevalence of drug use

The peak in the number of drugs was observed at the start of MDD (AD mean 7.4 SD ± 3.1, no AD mean 8.2 SD ± 3.1) (Table 1). Two years after the start of MDD, the number of drugs was slightly lower (AD 7.0 ± 3.2, no AD 7.7 ± 3.3) in both cohorts. At the start of MDD, the most commonly used drugs among persons with AD were antidementia drugs (84.8%), cardiovascular drugs (16.9–49.0% depending on drug group), paracetamol (35.9%) and antidepressants (33.7%) (Online resource Table 3). The same drugs were also the most commonly used in people with AD without MDD. The most frequently used drugs at the start of MDD in people without AD were cardiovascular drugs (22.5–66.0% depending on drug group), paracetamol (44.3%), proton-pump inhibitors (PPIs) (40.6%) and antidepressants (28.9%). The use of paracetamol (19.8%), PPIs (19.5%) and antidepressants (10.6%) was more common in AD persons compared to persons without AD.

**Fig. 2:**
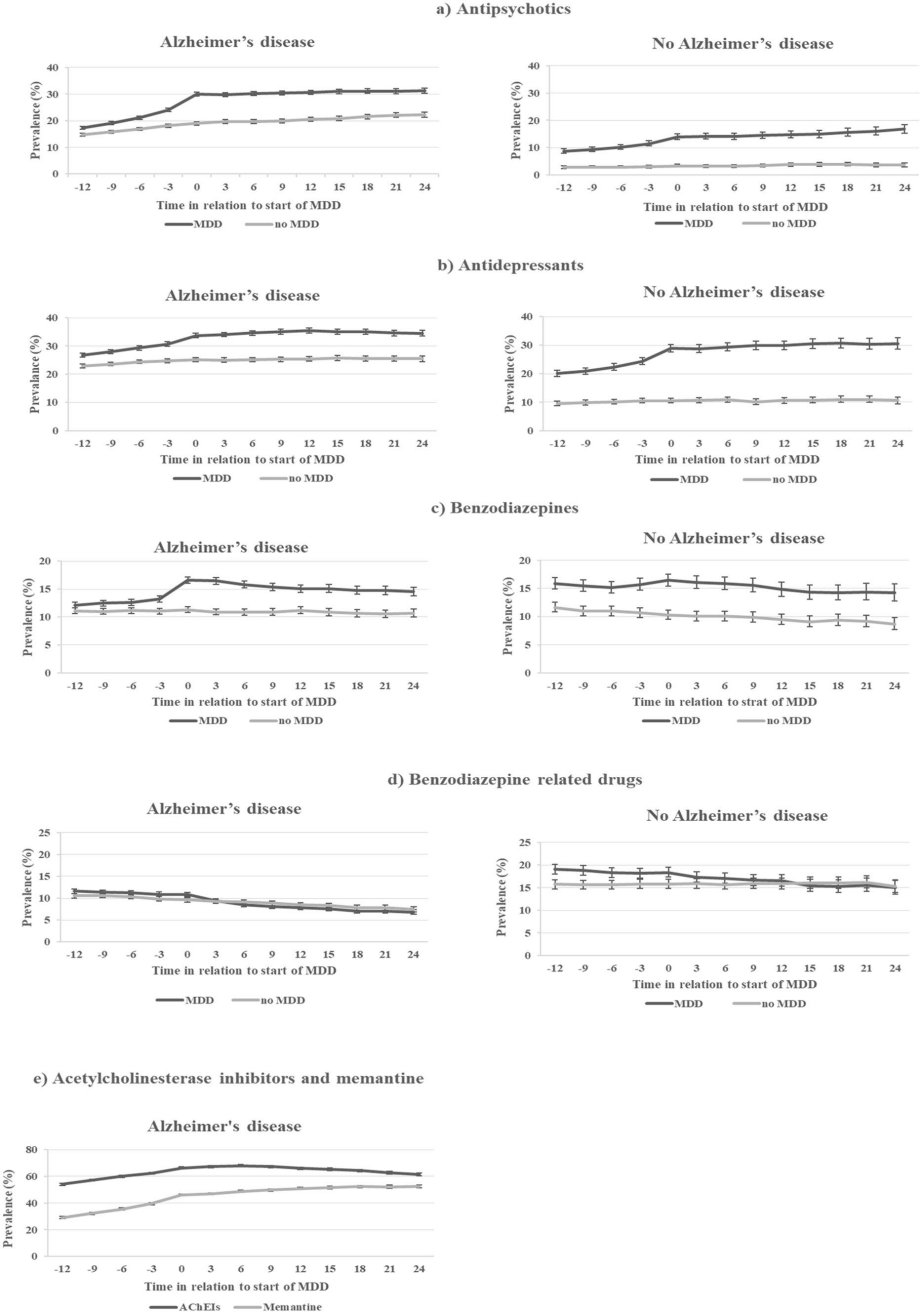
Prevalence of use of antipsychotics **a**), antidepressants **b**), benzodiazepines **c**) and benzodiazepine-related drugs **d**) in persons with and without Alzheimer’s disease (AD) and the prevalence of use of acetylcholinesterase inhibitors and memantine **e**) in persons with Alzheimer’s disease 12 months before the start of multi-dose dispensing (MDD) to 24 months after start of MDD. AChEIs, acetylcholinesterase inhibitors

Among AD persons, the use of antipsychotics, antidepressants and benzodiazepines increased around the start of MDD and the use remained on a higher level during the next 24 months compared to no-MDD (Fig. 2). Among persons without AD, the prevalence of antipsychotics and antidepressants also increased at the start of MDD compared to no-MDD. Memantine use increased among persons with AD in the start of MDD and it remained at a high level during the follow-up.

**Fig. 3:**
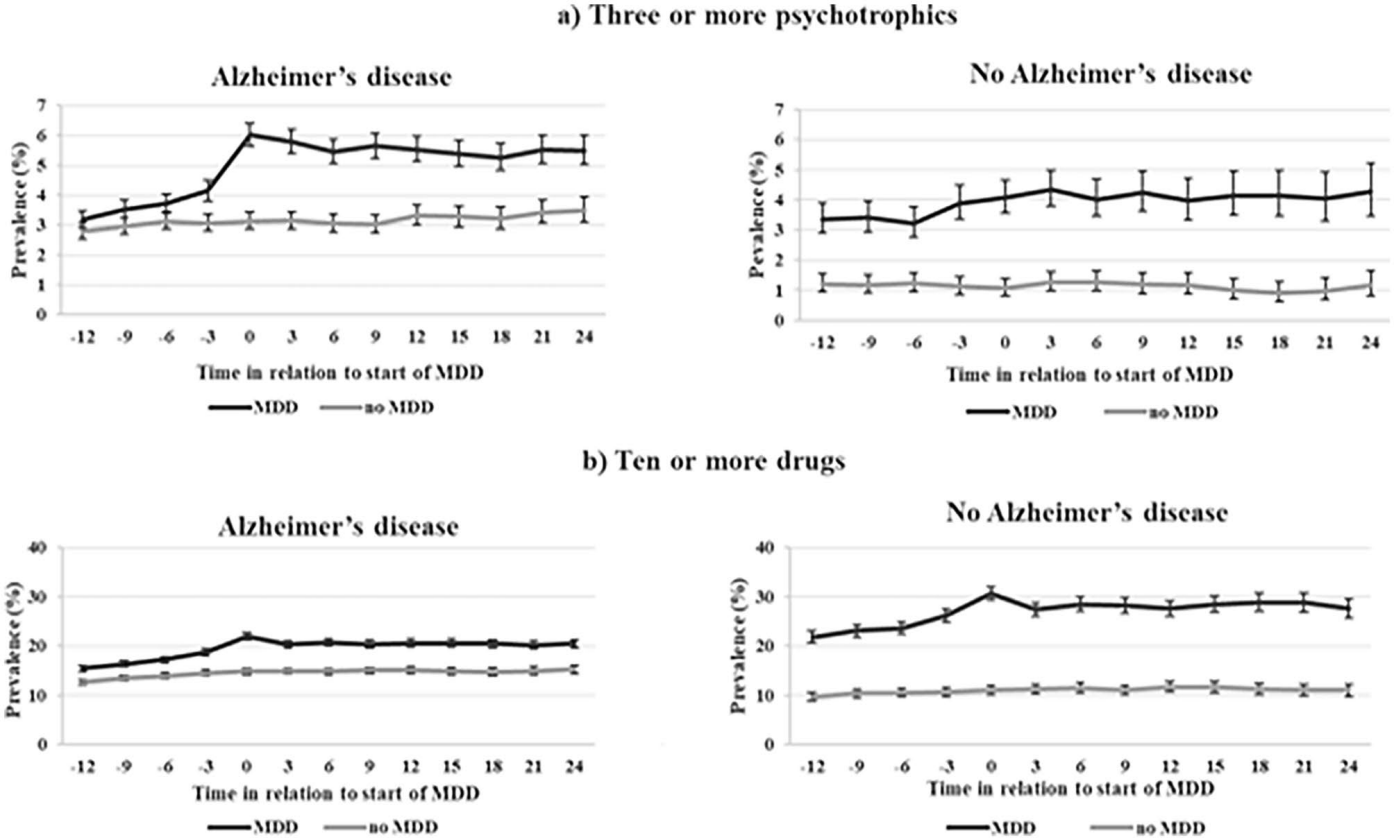
Prevalence of use of three or more psychotropics **a**) and ten or more drugs **b**) in persons with and without Alzheimer’s disease (AD) 12 months before the start of multi-dose dispensing (MDD) to 24 months after start of MDD

Among AD persons, the concomitant use of ≥ 3 psychotropics increased at the start of the MDD from 3.2% 12 months before (2.8% among no-MDD) to 6.0% at the start of MDD (3.1% among no-MDD) (Fig. 3). This use remained for 24 months with higher levels observed in AD persons with MDD (5.5%) compared to no-MDD (3.5%). Among AD persons, the use of ten or more drugs increased from 15.5% 12 months before to 21.9% at the start of MDD, and respectively from 21.8 to 30.5% in persons without AD.

Among AD persons at the start of MDD, the use of paracetamol and opioids (Online resource Fig. 1), loop diuretics (Online resource Fig. 2), PPIs and mirtazapine (Online resource Fig. 3) increased, and the use remained on a higher level during the next 24 months compared to no-MDD. The same phenomenon was seen also among persons without AD at the start of MDD in comparison to no-MDD. The use of benzodiazepine-related drugs (Fig. 2), NSAIDs (Online resource Fig. 1) and urinary antispasmodics (Online resource Fig. 3) had a decreasing trend among persons with AD in both MDD and no-MDD groups during the whole follow-up period.

## Discussion

To the best of our knowledge, this was the first study describing the use of drugs before and after the start of MDD among community dwellers with AD in comparison to persons without AD. Although MDD is aimed to improve pharmacotherapy, our study showed contrary effects. The use of antipsychotics, antidepressants, benzodiazepines and opioids and concomitant use of three or more psychotropic drugs and ten or more drugs increased at the start of MDD among persons with AD. In addition, the prevalence of memantine and paracetamol use increased at the start of MDD among persons with AD. The use of antipsychotics, antidepressants, opioids and ten or more drugs increased also among persons without AD. However, the use of benzodiazepine-related drugs, NSAIDs and urinary antispasmodics had a decreasing trend already before MDD and the same trend continued until the end of follow-up among persons with and without AD.

Among persons with AD, antipsychotics, antidepressants and benzodiazepines are used to treat neuropsychiatric symptoms (NPS) [16] and high use of antipsychotics and antidepressants has also been shown in a previous study [21]. Similar to our findings, previous studies reported an increase of three or more psychotropics use among persons utilizing MDD [8, 9]. This finding of increasing use of psychotropics is not in accordance to the guideline as psychotropics should be used only if nonpharmacological treatments were ineffective for treating NPS [16]. The Finnish Current Care Guideline recommends use of only one psychotropic drug at a time for the treatment of NPS among persons with cognitive disorders and the use should be assessed within 3–6 months. The evidence on efficacy of psychotropic drugs to NPS is inconsistent and due to increased risk of severe adverse drug effects and events, like falls, hip fractures, pneumonia, stroke and death, concomitant use of psychotropics should be avoided [22–26]. However, the guideline acknowledges that in specific situations of individual patients concomitant use is possible [16]. It might be that NPS and pharmacotherapy of these symptoms are one reason to start of MDD. The same may apply to increased use of memantine, as its main indication is NPS. Pain is common in persons with AD, with untreated pain potentially provoking NPS [27]. Increasing use of paracetamol and opioids might also be related to NPS.

Increase in benzodiazepine use may also be related to reimbursement issues. In MDD, large package sizes are favoured, and a person may have used smaller, non-reimbursed benzodiazepine packages before the MDD service. Thus, actual benzodiazepine use may have remained on the same level and MDD may represent just the transition from non-reimbursed smaller packages to reimbursed packages. MDD is a way to restrict the number of tablets of potential risk drugs that the patient has at home because the drugs are dispensed and administered only for 2 weeks, whereas ordinary prescriptions may be dispensed for 3 months.

Previous studies [8, 19] have reported increased number of drugs, which is similar to our findings. In our study, persons starting with MDD had more comorbid conditions, more inpatient hospital days 1 month before MDD and higher prevalence of excessive polypharmacy already 1 year before the start of MDD compared to persons without MDD. This is also seen in previous studies reporting higher number of comorbid conditions and healthcare contacts among persons who start MDD [9, 10]. It seems that MDD has been started for persons with a high burden of diseases. Based on our clinical experience, the need of MDD is assessed when a person starts to receive home care services or is moving to residential care. These changes are signs of worsening of function, diseases or symptoms.

Swedish studies found a higher prevalence of anticholinergics use among patients in MDD than without MDD [7, 8]. In our study, there were no differences in the use of anticholinergic urinary antispasmodics in AD population, but among persons without AD these drugs were more often used in persons with MDD than without MDD. This finding among persons with AD is encouraging as anticholinergics have potential pharmacodynamic interaction with acetylcholinesterase inhibitors [7, 28].

A major strength of this study is the nationwide cohort of community-dwelling individuals with clinically verified diagnosis of AD and long-term follow-up of their drug use through registers. We used the PRE2DUP method for drug use modelling, which takes into account periods in hospital/institutional care when drugs are provided by the caring unit and not recorded in the Prescription Register.

Limitations of the study are related to register-based nature of data. Small packages of some drugs, such as benzodiazepines, were not reimbursed and thus, not included in the data. The data do not include drug use in institutions or over-the-counter drugs which could lead to an underestimation of the drug use. The lack of information about disease severity and indication for drug use are also limitations in our study.

## Conclusions

Our findings on the increasing use of antipsychotics, antidepressants, opioids and ten or more drugs at the start of MDD among persons with and without AD are concerning. The use of benzodiazepines and three or more psychotropic drugs increased at start of MDD in persons with AD. It seems that MDD is initiated when number of drugs increases, and management of medication becomes harder. The MDD should be developed to improve drug safety by regular interprofessional medication assessment before the start of MDD and on regular basis after that.

## Supplementary information

Below is the link to the electronic supplementary material.

Supplementary file1 (PPTX 101 KB)

Supplementary file2 (PPTX 219 KB)

Supplementary file3 (PPTX 126 KB)

Supplementary file4 (DOCX 22 KB)

## Data Availability

Not applicable.
