# Supplementary figures and images for: Automated multi-dose dispensing in persons with and without Alzheimer’s disease—impacts on pharmacotherapy
Source: Eur J Clin Pharmacol. 2021 Nov 27;78(3):513–21. doi: 10.1007/s00228-021-03258-y (PMC8818643; doi:10.1007/s00228-021-03258-y)

## Slide 1
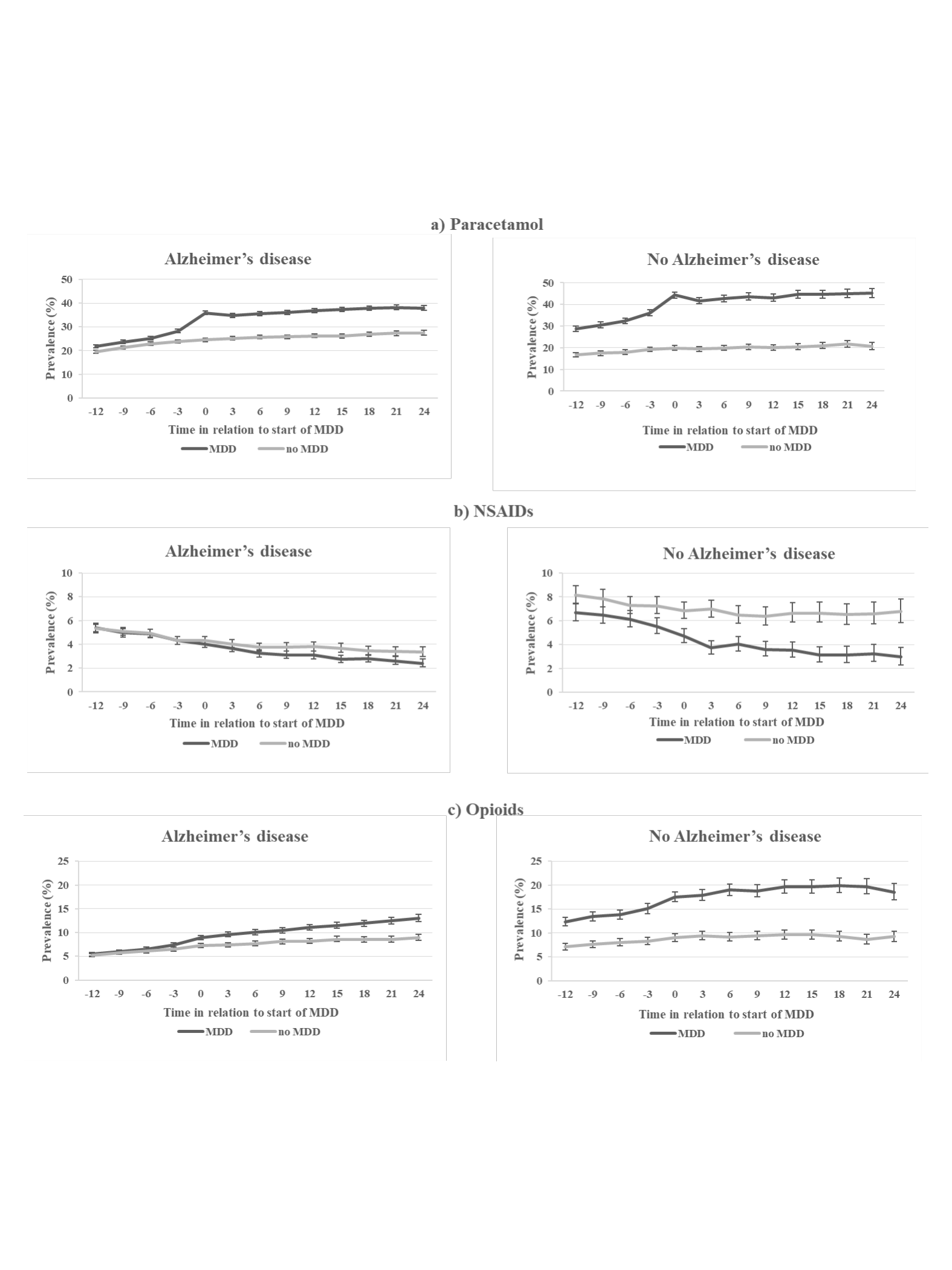

Supplement: Supplementary file 1 — Supplementary file1 (PPTX 101 KB) [file 228_2021_3258_MOESM1_ESM.pptx]

## Slide 1
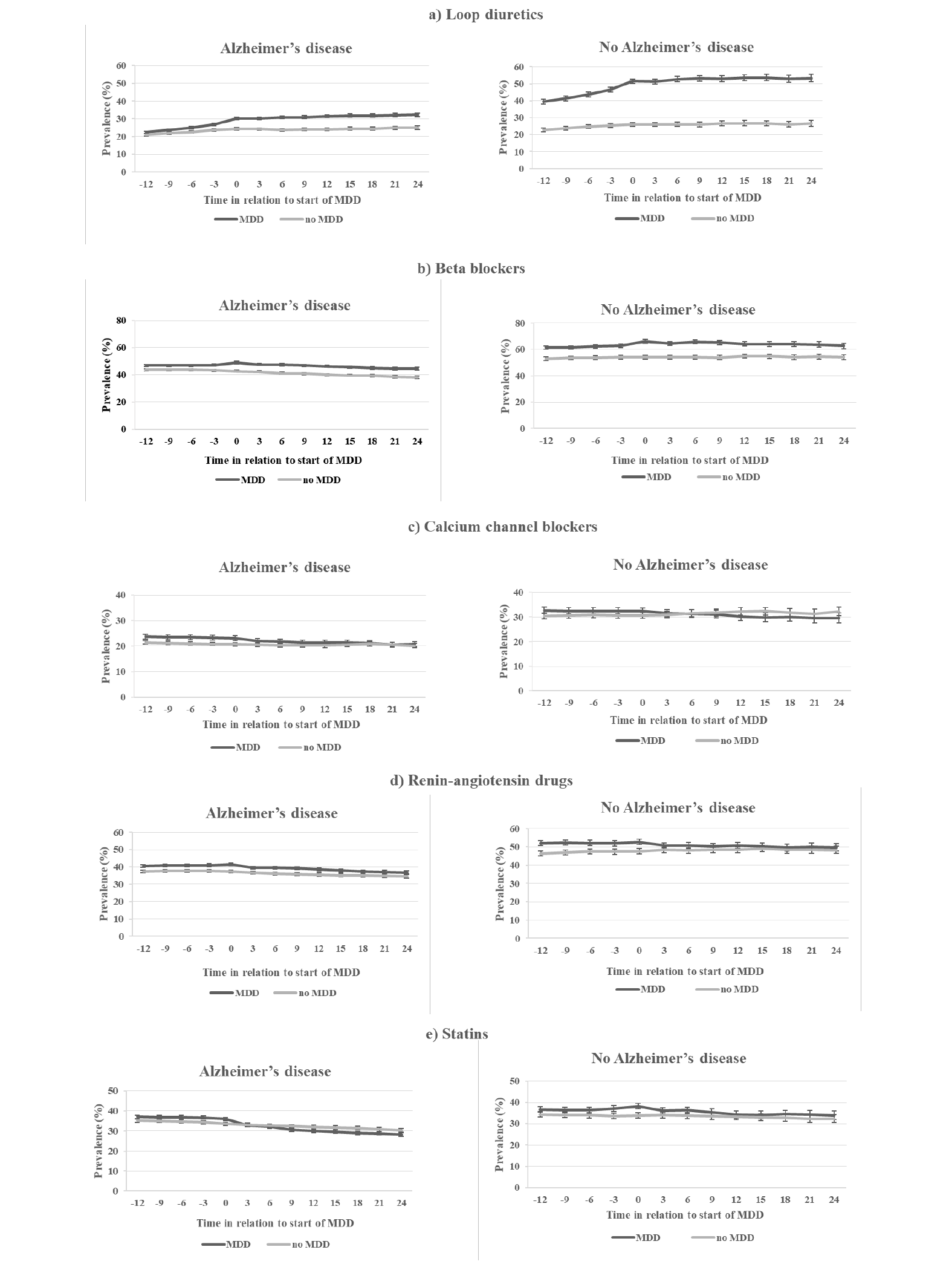

Supplement: Supplementary file 2 — Supplementary file2 (PPTX 219 KB) [file 228_2021_3258_MOESM2_ESM.pptx]

## Slide 1
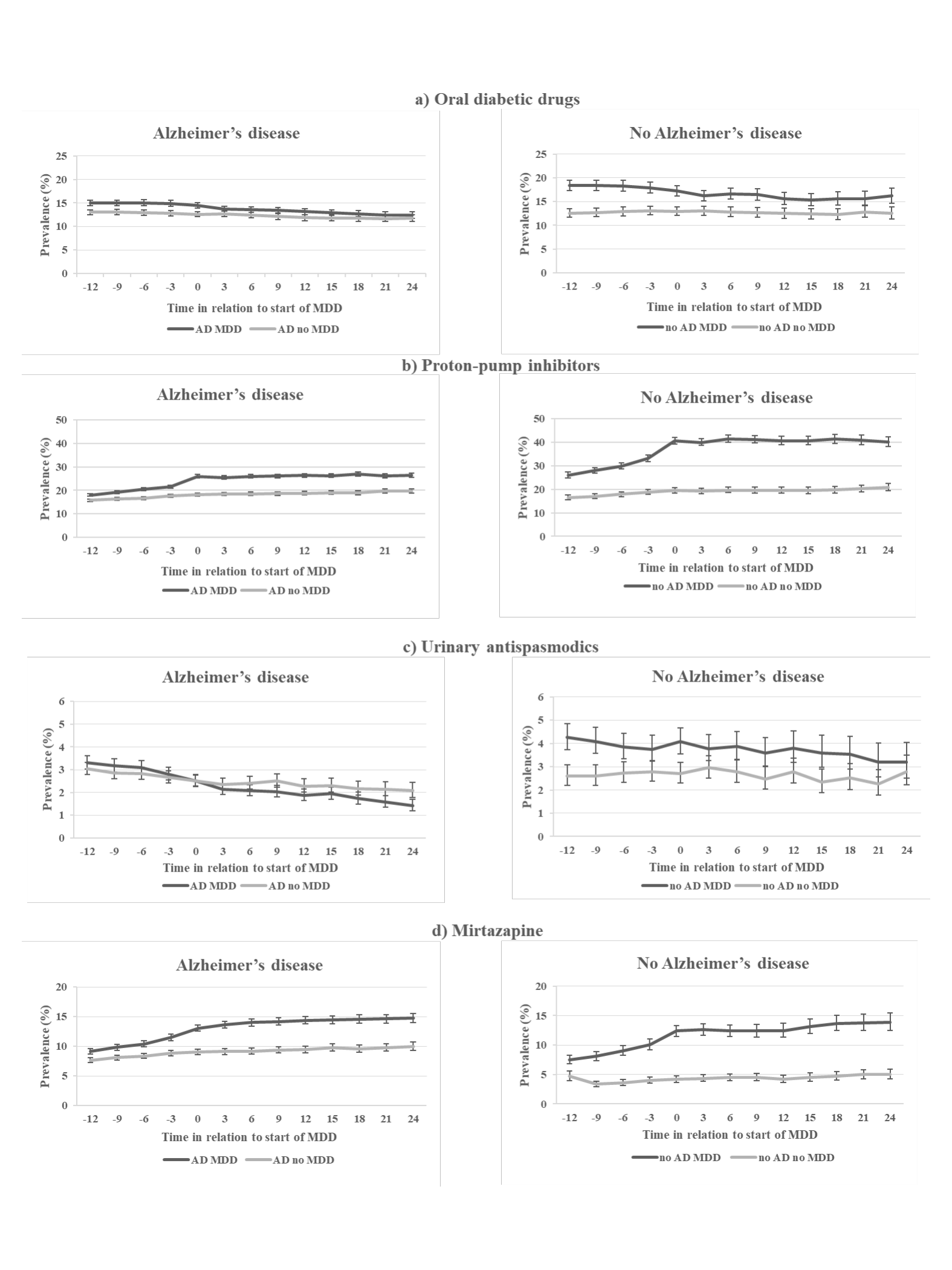

Supplement: Supplementary file 3 — Supplementary file3 (PPTX 126 KB) [file 228_2021_3258_MOESM3_ESM.pptx]
