# Supplementary material for: Automated multi-dose dispensing in persons with and without Alzheimer’s disease—impacts on pharmacotherapy
Source: Eur J Clin Pharmacol. 2021 Nov 27;78(3):513–21. doi: 10.1007/s00228-021-03258-y (PMC8818643; doi:10.1007/s00228-021-03258-y)
Supplement: Supplementary file 4 — Supplementary file4 (DOCX 22 KB) [file 228_2021_3258_MOESM4_ESM.docx]

ONLINE RESOURCE TABLES 1-3

Online resource Table 1. Drugs analyzed according MDD and ATC-codes

| **Drug/drug class** | **ATC-code** |
| --- | --- |
| Antidementia drugs | N06D |
| Benzodiazepines | N05BA |
| Benzodiazepine related drugs (zopiclone and zolpidem) | N05CF |
| Antipsychotics | N05A |
| Antidepressants (categorized as selective serotonin reuptake inhibitors, SSRIs, mirtazapine and other antidepressants) | N06AB, N06AX11, N06AG02, N06AX03, N06AX05, N06AX12, N06AX18, N06AX22, N06AX26 |
| Opioids | N02A |
| Paracetamol | N02BE01 |
| Nonsteroidal inflammatory drugs (NSAIDs excluding glucosamine) | M01A excluding M01AX05 |
| Oral antidiabetics | A10B |
| Loop diuretics | C03CA |
| Other diuretics | C03 |
| Beta blockers | C07 |
| Calcium channel blockers | C08 |
| Renin angiotensin group | C09A, C09B, C09C, C09D |
| Statins | C10AA, C10BA |
| Antiepileptics | N03A |
| Proton pump inhibitors (PPIs) | A02BC |
| Urinary antispasmodics excluding mirabegron. | G04BD excluding G04BD12 |

Online resource Table 2. ICD-codes of comorbidities that persons had at the time of start of multi-dose dispensing (the start of follow-up).

| **Hypertension** | Hospitalization (ICD-10: I10‒I15) or special reimbursement (classification number 205) | Diagnosed since 1996 until the start of follow-up (hospitalization); or since 1972 until the start of follow-up (special reimbursement) | FCR, SRR |
| --- | --- | --- | --- |
| **Coronary artery disease** | Hospitalization (ICD-10 I20-I25; NOMESCO FNA, FNC, FNE, FNG00, FNG10, FN1AT, FN1BT, FN1YT) or special reimbursement of coronary artery disease (classification numbers 206, 213, 280) | Diagnosed since 1996 until the start of follow-up (hospitalization); or since 1972 until the start of follow-up (special reimbursement) | FCR, SRR |
| **Chronic heart failure** | Hospitalization (ICD-10: I42‒43, I50, I11.0) or special reimbursement (classification number 201) | Diagnosed since 1996 until the start of follow-up (hospitalization); or since 1972 until the start of follow-up (special reimbursement) | FCR, SRR |
| **Atrial fibrillation** | Hospitalization (ICD-10: I48) | Diagnosed since 1996 until the start of follow-up | FCR |
| **Cardiovascular disease** | Hypertension, coronary artery disease, chronic heart failure or atrial fibrillation diagnosed (as defined above) | Diagnosed since 1996 until the start of follow-up (hospitalization); or since 1972 until the start of follow-up (special reimbursement) | FCR, SRR |
| **History of stroke** | ICD-10: I60‒I64, I69;  ICD-9: 430-432, 4330A, 4331A, 4339A, 4349A, 4340A, 4341A, 4360;  ICD-8: 430-434 | Diagnosed since 1972 until the start of follow-up | FCR |
| **Any psychiatric disorder** | Schizophrenia, bipolar disorder or depression;  Hospitalization (ICD-10: F20-F29, F30-F34, F38-F39) | Diagnosed since 1972 until 5 years prior to the diagnosis of AD | FCR |
| **Substance abuse** | Hospitalization (ICD-10: K86.0, F10-F19, K70; ICD-9: 291, 292, 2940A, 2948X, 303, 304, 305, 5770D-F, 5771C, 5771D, 5710A, 5711A, 5712A, 5713X; ICD-8: 291, 303, 304, 57700-57708, reason for admission codes 33\|71\|72\|73\|74\|75), drug use for addiction (ATC: N07BB, N07BC) | Diagnosed since 1972 until the start of follow-up in FCR, or purchased ever since 1995 before the start of follow-up in the PR | FCR |
| **Any cancer** | Hospitalization (ICD-10: C00-C97, Z85; ICD-9: 140-195, 2730, 2733, V1046, 200-208) | Diagnosed since 1987 until the start of follow-up | FCR |
| **Asthma/COPD** | Hospitalization (ICD-10: J44-J46) or special reimbursement (classification number 203) | Diagnosed since 1996 until the start of follow-up in the FCR or since 1972 until the start of follow-up in the SRR | FCR, SRR |
| **Rheumatoid arthritis** | Hospitalization (ICD-10: M05, M06, M45) or special reimbursement (classification number 202) | Diagnosed since 1996 until the start of follow-up in the FCR or since 1972 until the start of follow-up in the SRR | FCR, SRR |
| **Epilepsy** | Special reimbursement (classification number 111) | Diagnosed since 1972 until the start of follow-up | SRR |
| **Diabetes** | Diabetes medication (ATC: A10), or special reimbursement for diabetes (classification number 103) | At least one purchase prior to the start of follow-up in the PR or diagnosed since 1972 until the start of follow-up in the SRR | PR, SRR |
| **Osteoporosis** | Bisphosphonate use M05BA, M05BB | Ever (since 1995) before the start of follow-up | PR |
| **Hypothyreosis** | Special reimbursement for hypothyreosis or drug use (H03) | Ever (since 1995) before the start of follow-up (drugs), since 1972 (special reimbursement) | SRR, PR |
| **Glaucoma** | Glaucoma drug use S01E | Ever (since 1995) before the start of follow-up | PR |

FCR: Finnish Care register for Health Care, SRR: Special Reimbursement Register, PR: Prescription Register

Online resource table 3. Prevalence of drug use of the study cohort in the start of MDD according to Alzheimer’s disease (AD) and multi-dose dispensing (MDD) status

|  | **Persons with AD** | |  | **Persons without AD** | |  |
| --- | --- | --- | --- | --- | --- | --- |
|  | **MDD**  **N=14724^a^**  **N (%)** | **no MDD**  **N=14542**  **N (%)** | **p-value** | **MDD**  **N=4961**  **N (%)** | **no MDD**  **N=5093**  **N (%)** | **p-value** |
| Antidementia drugs | 12 493 (84.8) | 11 982 (82.4) | <0.001 | 59 (1.2) | 12 (0.2) | <0.001 |
| AChEIs | 9770 (66.4) | 9534 (65.6) | 0.153 | 53 (1.1) | 12 (0.2) | <0.001 |
| Memantine | 6771 (46.0) | 5551 (38.2) | <0.001 | 15 (0.3) | 1 (0.0) | <0.001 |
| Antipsychotics | 4405 (29.9) | 2765 (19.0) | <0.001 | 695 (14.0) | 172 (3.4) | <0.001 |
| All antidepressants | 4959 (33.7) | 3645 (25.1) | <0.001 | 1435 (28.9) | 538 (10.6) | <0.001 |
| SSRIs | 2961 (20.1) | 2221 (15.3) | <0.001 | 733 (14.8) | 255 (5.0) | <0.001 |
| Mirtazapine | 1925 (13.1) | 1311 (9.0) | <0.001 | 614 (12.4) | 221 (4.3) | <0.001 |
| Other | 469 (3.2) | 353 (2.4) | <0.001 | 238 (4.8) | 103 (2.0) | <0.001 |
| BZDs | 2441 (16.6) | 1639 (11.3) | <0.001 | 815 (16.4) | 524 (10.3) | <0.001 |
| Long-acting BZDs | 89 (0.6) | 90 (0.6) | 0.874 | 66 (1.3) | 58 (1.1) | 0.384 |
| BZD related drugs | 1595 (10.8) | 1399 (9.6) | 0.001 | 911 (18.4) | 805 (15.8) | 0.001 |
| Analgetics |  |  |  |  |  |  |
| Opiods | 1305 (8.9) | 1051 (7.2) | <0.001 | 869 (17.5) | 457 (9.0) | <0.001 |
| NSAIDs | 593 (4.0) | 629 (4.3) | 0.203 | 234 (4.7) | 349 (6.9) | <0.001 |
| Paracetamol | 5292 (35.9) | 3562 (24.5) | <0.001 | 2199 (44.3) | 1010 (19.8) | <0.001 |
| Urinary antispasmodics | 368 (2.5) | 366 (2.5) | 0.924 | 202 (4.1) | 138 (2.7) | <0.001 |
| Cardiovascular drugs |  |  |  |  |  |  |
| Loop diuretics | 4441 (30.2) | 3527 (24.3) | <0.001 | 2555 (51.5) | 1319 (25.9) | <0.001 |
| Other diuretics | 6207 (42.2) | 5353 (36.8) | <0.001 | 3126 (63.0) | 2291 (45.0) | <0.001 |
| Beta blockers | 7209 (49.0) | 6208 (42.7) | <0.001 | 3276 (66.0) | 2757 (54.1) | <0.001 |
| Calcium channel blockers | 3390 (23.0) | 3001 (20.6) | <0.001 | 1604 (32.3) | 1560 (30.6) | 0.066 |
| ACE inhibitors | 3645 (24.8) | 3176 (21.8) | <0.001 | 1523 (30.7) | 1237 (24.3) | <0.001 |
| ATR blockers | 2493 (16.9) | 2297 (15.8) | 0.009 | 1118 (22.5) | 1208 (23.7) | 0.160 |
| Statins | 5291 (35.9) | 4867 (33.5) | <0.001 | 1896 (38.2) | 1724 (33.9) | <0.001 |
| Other |  |  |  |  |  |  |
| Oral diabetes drugs | 2136 (14.5) | 1834 (12.6) | <0.001 | 856 (17.3) | 659 (12.9) | <0.001 |
| PPIs | 3821 (26.0) | 2650 (18.2) | <0.001 | 2012 (40.6) | 994 (19.5) | <0.001 |
| Antiepileptics | 1083 (7.4) | 775 (5.3) | <0.001 | 524 (10.6) | 186 (3.7) | <0.001 |
| ≥10 Drugs | 3227 (21.9) | 2154 (14.8) | <0.001 | 1515 (30.5) | 562 (11.0) | <0.001 |
| ≥3 Psycho- trophic drugs | 886 (6.0) | 455 (3.1) | <0.001 | 202 (4.1) | 54 (1.1) | <0.001 |

^a^Prevalence was assessed in 2-week time windows. Persons who were in hospital/institutional care for more than 5 days of the of the 2-week time window were excluded: N=880 AD in MDD, N=1062 AD no-MDD, N=263 in non-AD MDD and N=131 in non-AD no-MDD.

AChEIs =acetylcholinesterase inhibitors, ACE=Angiotensin-converting enzyme, ATR= Angiotensin receptor blocker, SSRIs= selective serotonine reuptake inhibitors, BZDs = benzodiazepines, PPIs = proton-pump inhibitors
